# Metformin Loaded Zein Polymeric Nanoparticles to Augment Antitumor Activity against Ehrlich Carcinoma via Activation of AMPK Pathway: D-Optimal Design Optimization, In Vitro Characterization, and In Vivo Study

**DOI:** 10.3390/molecules29071614

**Published:** 2024-04-03

**Authors:** Yasmina Elmahboub, Rofida Albash, Mira Magdy William, Amal H. Rayan, Najat O. Hamed, Mona S. Ousman, Nahed A Raslan, Shaimaa Mosallam

**Affiliations:** 1 Department of Pharmaceutics, College of Pharmaceutical Sciences and Drug Manufacturing, Misr University for Science and Technology, Giza 12585, Egypt; yasmina.elmahboub@must.edu.eg; 2 Department of Biochemistry, Faculty of Pharmacy, October 6 University, Giza 12585, Egypt; 3 Department of Medical Education, College of Medicine, AlMaarefa University, Diriyah, Riyadh 13713, Saudi Arabia; 4 Department of Pharmaceutical Sciences, Faculty of Pharmacy, AlMaarefa University, Diriyah, Riyadh 13713, Saudi Arabia; nhamed@um.edu.sa; 5 Emergency Medical Services, College of Applied Sciences, AlMaarefa University, Diriyah, Riyadh 13713, Saudi Arabia; mousman@um.edu.eg; 6 Department of Pharmacology and Toxicology, Faculty of Pharmacy (Girls), Al-Azhar University, Cairo 11651, Egypt; nahedraslan461@gmail.com; 7 Clinical Pharmacy Program, College of Health Sciences and Nursing, Al-Rayan Colleges, Medina 42541, Saudi Arabia; 8 Department of Pharmaceutics and Industrial Pharmacy, Faculty of Pharmacy, October 6 University, Giza 12585, Egypt; shaimaamosallam@o6u.edu.eg

**Keywords:** metformin, zein nanoparticles, breast cancer, D-optimal design, AMPK

## Abstract

Metformin (MET), an antidiabetic drug, is emerging as a promising anticancer agent. This study was initiated to investigate the antitumor effects and potential molecular targets of MET in mice bearing solid Ehrlich carcinoma (SEC) as a model of breast cancer (BC) and to explore the potential of zein nanoparticles (ZNs) as a carrier for improving the anticancer effect of MET. ZNs were fabricated through ethanol injection followed by probe sonication method. The optimum ZN formulation (ZN8) was spherical and contained 5 mg zein and 30 mg sodium deoxycholate with a small particle size and high entrapment efficiency percentage and zeta potential. A stability study showed that ZN8 was stable for up to three months. In vitro release profiles proved the sustained effect of ZN8 compared to the MET solution. Treatment of SEC-bearing mice with ZN8 produced a more pronounced anticancer effect which was mediated by upregulation of P53 and miRNA-543 as well as downregulation of NF-κB and miRNA-191-5p gene expression. Furthermore, ZN8 produced a marked elevation in pAMPK and caspase-3 levels as well as a significant decrease in cyclin D1, COX-2, and PGE2 levels. The acquired findings verified the potency of MET-loaded ZNs as a treatment approach for BC.

## 1. Introduction

One of the most common cancers in women is breast cancer (BC). Some cases of BC may not respond well to standard treatment [1]. It is worth mentioning that Metformin (MET) reduces the development of resistance in BC cells [2]. MET is a biguanide compound that is known for decreasing glucose levels, and stimulates insulin sensitivity and glucose uptake and inhibits hepatic gluconeogenesis in diabetic patients [1]. The antitumor effects of MET are attributed to both direct (insulin-independent) as well as indirect (insulin-dependent) actions. These direct effects are linked to liver kinase B1 (LKB1)-mediated activation of AMP-activated protein kinase (AMPK) and suppression of mammalian target of rapamycin (mTOR) signaling and protein synthesis in cancer cells. The indirect effects come from its insulin-lowering action because insulin possesses prosurvival and mitogenic properties [2]. The cellular energy sensor and regulator AMPK is crucial for lipid and protein metabolism regulation in response to fluctuations in fuel availability [3,4]. Numerous studies have shown that AMPK blocks the anabolic pathways that stimulate cell growth, including phospholipid, fatty acid, and protein as well as ribosomal RNA synthesis [5]. Since tumor cells exhibit a higher energy demand due to their capacity for rapid growth and division, AMPK is expected to prevent the proliferation of cancer cells [1].

The utilization of polymeric nanoparticles enables the creation of cancer medications with better bioavailability. Nanoparticles can enter the tumor cells via the improved permeability and retention effect because of their small particle size and large surface area [2]. Zein is a major storage protein, and is made up of more than 50% non-polar amino acid residues [6]. On the contrary, zein exhibits a specific polarity because of its high glutamine concentration. The presence of both hydrophobic and hydrophilic regions in zein enables it to acquire an amphiphilic character [7]. Since the hydrophobic regions are dominating in the structure of zein, it combines to form colloidal particles that can retain lipophilic medicines, whilst the polar areas interact with water-soluble molecules [8].

Zein nanoparticles (ZNs) have sparked great attention because of their biocompatibility, ability to retain either hydrophilic or hydrophobic drugs, and high stability. Additionally, the large surface area and small size of colloids can facilitate their interaction with aqueous media, which favors the localization of the entrapped compounds inside the tumor through their improved permeation and retention effects. Furthermore, ZNs can influence how well cancer cells’ drug efflux pumps work, reducing the level of multidrug resistance [9].

Previous studies had verified the potential of ZNs as a carrier for improving the activity of anticancer agents. Shinde et al. illustrated the capability of entrapping carvacrol- ZNs for enhanced anticancer activity against colon cancer [10]. Another study by Liu et al. demonstrated the potency of hyaluronic acid–zein core-shell nanoparticles for improving the anticancer effect of curcumin [11].

To the best of our knowledge, the encapsulation of MET into ZNs to boost its anticancer effect has not been previously investigated. We are the first to evaluate the effect of MET-loaded ZNs on activation of AMPK pathway as well as the modulation of miR-191-5p and miR-543 as a potential mechanism for their anticancer effect in BC. Hence, the objective of the present study was to explore the potential molecular targets as well as beneficial effects of implementing MET-loaded ZNs as an effective BC therapy.

## 2. Results

### 2.1. Analysis of D-Optimal Design

**Figure 1:**
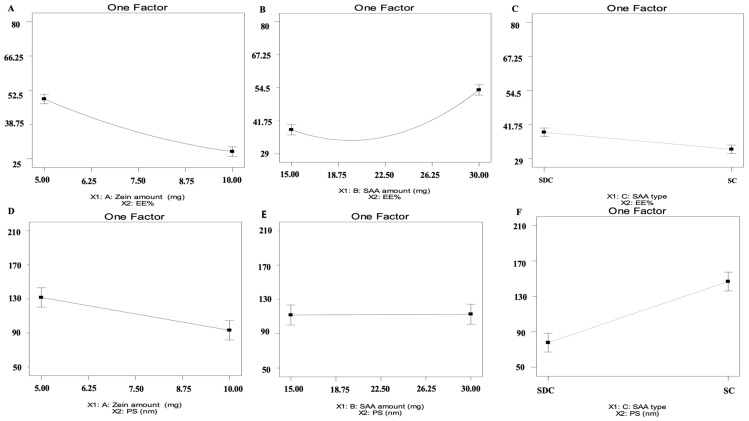
Effect of formulation variables on EE% of MET-loaded ZNs (**A**–**C**), effect of formulation variables on PS of ZNs (**D**–**F**). Abbreviation: EE%: entrapment efficiency percentage, PS: particle size, MET: metformin, and ZNs: zein nanoparticles.

**Figure 2:**
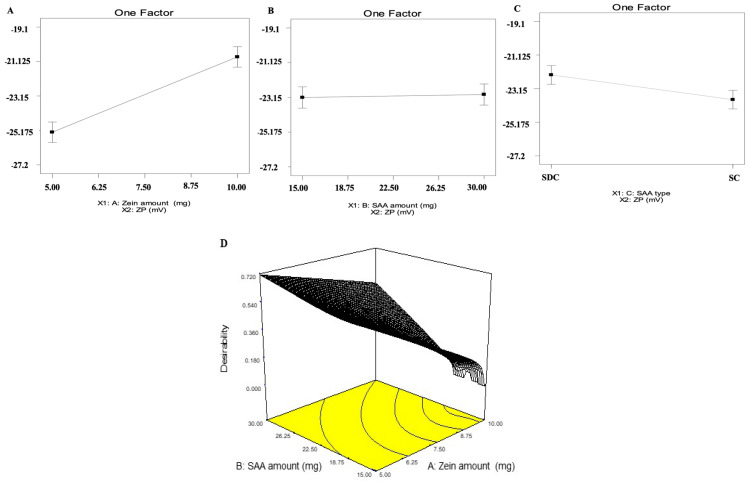
Effect of formulation variables on ZP of MET-loaded ZNs (**A**–**C**), (**D**) desirability figure of the optimum ZNs (ZN8). Abbreviation: ZP: zeta potential, MET: metformin, ZNs: zein nanoparticles.

The fabricated ZNs were optimized utilizing D-optimal design applying Design-expert^®^ (Version 7, State-Ease Inc., Minneapolis, MN, USA), resulting in 15 experimental trials with the exclusion of the non-significant response polydispersity index (PDI). For entrapment efficiency percent (EE%), the chosen model was quadratic, however it was 2FI for particle size (PS) and zeta potential (ZP). As shown in Table 1, all responses had ratios greater than four, which is the ideal ratio for adequate precision [12]. The predicted R^2^ values agreed with the adjusted R^2^ in all responses (Table 1). Regarding EE% values, it ranged from 28.79% ± 6.54 to 77.68% ± 1.28 (Table 2) as illustrated in Figure 1A–C. For zein amount (mg) (X_1_), it was found that by increasing the zein amount from 5 to 10 mg, the EE% decreased. Increasing the bile salt amount (X_2_) increases MET EE% into the ZNs. Moreover, by altering the bile salt type (X_3_), the EE% values increased by using sodium deoxycholate (SDC) compared to sodium cholate (SC). Further, PS values ranged from 59.69 ± 1.79 to 126.80 ± 2.46 nm (Table 2) as presented in Figure 1D–F. For zein amount (mg) (X_1_), the PS values decreased by increasing the zein amount. For bile salt amount (X_2_), the factor was not significant with a *p*-value of 0.9314. Regarding bile salt type (X_3_), it was perceived that SDC showed higher PS values than SC. Moreover, PDI values ranged from 0.34 ± 0.12 to 0.99 ± 0.01 as depicted in Table 2. All the inspected factors revealed a non-significant effect on the PDI of the systems. Subsequently, it was removed from optimization. Further, ZP ranged from −20.30 ± 0.32 to −26.90 ± 1.06 mV (Table 2) as shown in Figure 2A–C. It was observed that by increasing the zein amount (mg) (X_1_), ZP significantly decreased (*p* = 0.0054). For bile salt amount (X_2_) (*p* = 0.773), the factor was not significant. Regarding bile salt type (X_3_), it was observed that ZP values were augmented using SC compared to SDC.

### 2.2. Selection of the Optimum ZNs

The overall desirability of the optimum ZNs (ZN8) was 0.713, which was proposed to be prepared to employ 5 mg as zein amount (X_1_), bile salt amount of 30 mg (X_2_), and bile salt type SDC as bile salt type (X_3_). Hence, it was prepared and evaluated. The optimum ZN formulation (ZN8) was considered promising and determined to be used in further investigation.

### 2.3. Characterization of the Optimum ZNs

#### 2.3.1. Transmission Electron Microscope (TEM)

**Figure 3:**
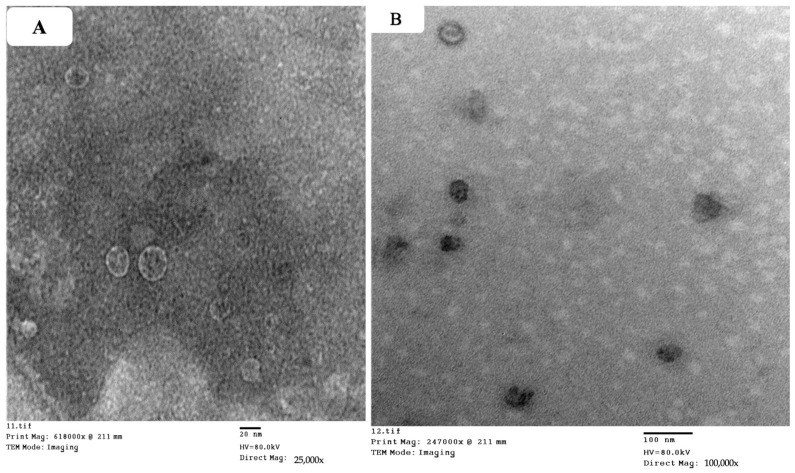
Transmission electron micrographs of optimum ZNs (ZN8) 25,000× (**A**), 100,000× (**B**). Abbreviation: ZNs: zein nanoparticles.

The structural evaluation revealed that the optimum ZN formulation (ZN8) had a homogeneous size distribution and was spherical (Figure 3). The PS of ZN8 as determined by Zetasizer agreed well with TEM findings [13].

#### 2.3.2. In Vitro Release Study

**Figure 4:**
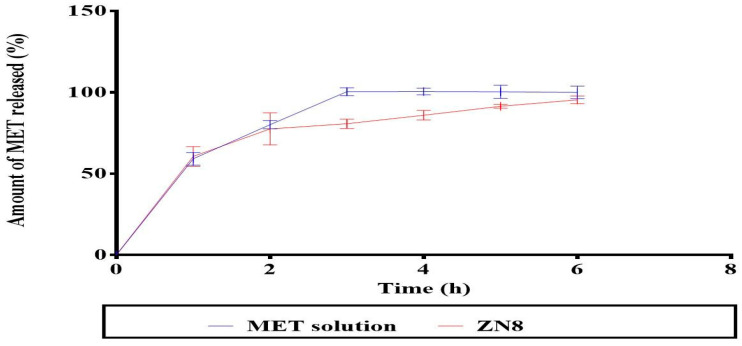
In vitro release profile of the MET solution and the optimum ZNs (ZN8). Abbreviation: MET; metformin, ZNs: zein nanoparticles.

Figure 4 shows that the release profile from ZN8 was initially rapid and subsequently significantly (*p* = 0.001) declined in a sustained manner compared to the MET solution, since MET was totally released from MET solution after three hours.

#### 2.3.3. Effect of Short-Term Storage

The values of stored ZN8 are 79.87% ± 0.1, 57.98 ± 3.90 nm, −24.0 ± 0.90 mV, and 0.53 ± 0.001. Regarding EE%, PS, ZP, and PDI, there was no statistical difference between the freshly fabricated ZN8 and those that had been kept. Furthermore, ZN8 external structure is unaltered during the storage period.

#### 2.3.4. Differential Scanning Calorimetry

**Figure 5:**
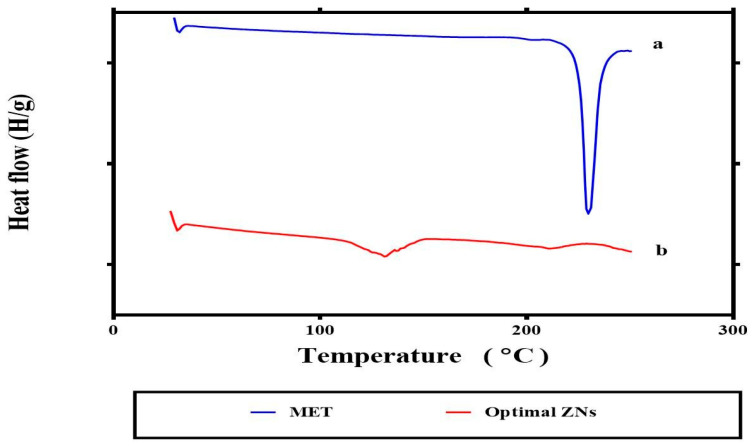
DSC thermogram of MET (**a**) and optimum ZNs (ZN8) (**b**). Abbreviation: MET: metformin, ZNs: zein nanoparticles.

Figure 5 represents the fact that the sharp endothermic peak at 221.32 °C may be due to the melting of MET [14]. For ZN8, the distinctive peak (Figure 5b) of MET completely disappeared, possibly indicating that MET was encapsulated inside the ZNs.

### 2.4. In Vivo Study

#### 2.4.1. Effect on Survival Rate and Tumor Volume

**Figure 6:**
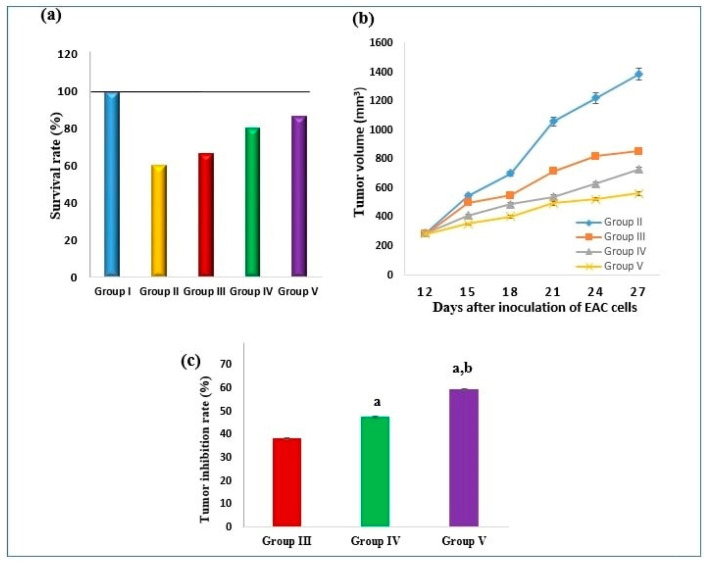
Effect of MET and ZN8 treatment of SEC-bearing mice on (**a**) survival rate, (**b**) tumor volume, and (**c**) tumor inhibition rate. Notes: Data are expressed as mean ± SEM. a: significant from SEC-bearing mice receiving MET, b: significant from SEC-bearing mice receiving ZN8.

The survival rate was 60% in the SEC control group, 66.7% in the MET treated group, 80% in the optimum MET-loaded ZNs (ZN8) treated group, and 86.7% in 5-flourouracil (5-FU) treated group (Figure 6a). On the 12th day, the tumor volume in the SEC control group was 289 ± 6.9 mm^3^ and increased gradually until reaching 1386 ± 42 mm^3^ on the 27th day. Conversely, treatment of SEC-bearing mice with MET, ZN8, and 5-FU exhibited a significant reduction in the tumor volume (*p* ˂ 0.05) in comparison to the SEC control group. On the 27th day, the tumor volume was 855.5 ± 17.4 mm^3^ in the MET treated group, 727.6 ± 18.3 mm^3^ in ZN8 treated group, and 562.2 ± 13 mm^3^ in 5-FU treated group (Figure 6b). In terms of tumor inhibition rate (TIR), treatment with MET exerted 38.3% versus 47.5% in ZN8 treated group and 59.4% in 5-FU treated group (Figure 6c).

#### 2.4.2. Effect on Tumor P53, NF-κB Gene Expression, and pAMPK Level

As shown in Table 3, P53 gene expression was downregulated in the SEC control group by 33.7% in comparison to the normal control group. However, treatment of SEC-bearing mice with MET, ZN8, and 5-FU produced a significant upregulation in P53 gene expression by 1.7-, 11.6-, and 4.8-fold, respectively, compared to the SEC control group with ZN8 treatment producing the most pronounced effect.

A significant increase (3.3-fold) in nuclear factor kappa-B (NF-κB) expression level was obtained in the SEC control group in comparison to normal control animals. In contrast, MET, ZN8, and 5-FU treatment significantly reduced NF-κB expression levels by 16.1%, 54.4%, and 31.7%, respectively, when compared to the SEC control group (Table 3) with ZN8 treatment producing the most pronounced effect.

The AMPK signaling pathway mediates the anticancer effects of MET. Thus, the current study examined whether MET may activate AMPK via phosphorylated adenosine monophosphate activated protein Kinase (pAMPK) level measurement. It was reduced in the SEC control group by 47.8% as compared to the normal control group (Table 3). MET, ZN8, and 5-FU administration significantly increased pAMPK levels by 1.4-, 1.6-, and 1.7-fold, respectively, relative to the SEC control group.

#### 2.4.3. Effect on Tumor Cyclin D1 and Caspase-3 Levels

As shown in Table 4, cyclin D1 level, a key regular of the G1/S transition, was markedly increased in the SEC control group by 7.4-fold compared to the normal control group. However, when SEC-bearing mice were treated with MET, ZN8, and 5-FU, cyclin D1 decreased by 28.9%, 67.5%, and 80.5%, respectively, compared to the SEC control group. Table 4 revealed that Ehrlich ascites carcinoma (EAC) cells inoculation in mice was associated with the suppression of apoptosis as reflected by the significant reduction of the pro-apoptotic activator caspase-3 level by 92.63% when compared to normal control. In contrast, caspase-3 level was significantly elevated in MET, ZN8, and 5-FU treated mice by 3.6-, 6.2-, and 8.2-fold, respectively, compared to the SEC control group.

#### 2.4.4. Effect on Tumor COX-2 and PGE2 Levels

The current work explored whether MET could inhibit cyclooxygenase-2 (COX 2) expression and prostaglandin E2 (PGE2) production. The levels of both COX-2 and PGE2 were markedly elevated in the SEC control group by 3.5- and 8.9-fold, respectively, as compared to the normal control group (Table 4). In contrast, treatment of SEC-bearing mice with MET, ZN8, and 5-FU produced a decrease in COX-2 level by 22.3%, 47.8%, and 60.1%, respectively, compared to the SEC control group. Similarly, PGE2 level was reduced in SEC-bearing mice following MET, ZN8, and 5-FU treatment by 31.7%, 74.8%, and 83.6, respectively, in comparison with the SEC control group (Table 4).

#### 2.4.5. Effect on miRNA-191-5p and miRNA-543

**Figure 7:**
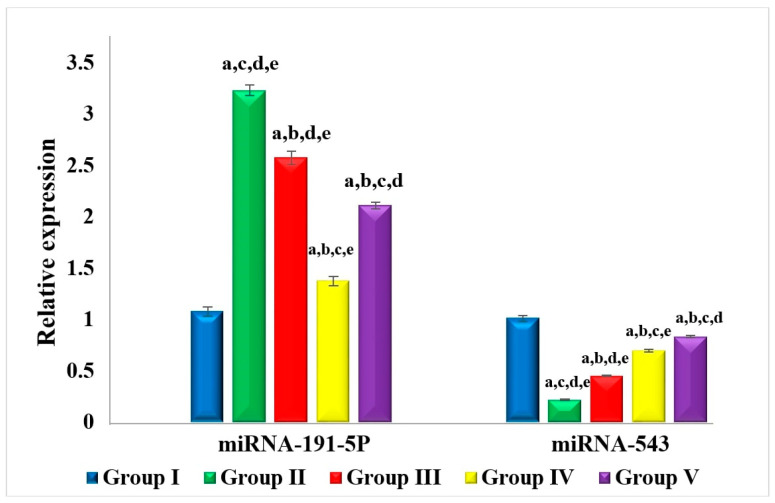
Effect of MET and ZN8 treatment of SEC-bearing mice on miRNA-191-5p and miRNA-543. Notes: Data are expressed as mean ± SEM. (a) statistically significant from normal controls. (b) Statistically significant from SEC control. (c) Statistically significant from SEC-bearing mice receiving MET. (d) Statistically significant from SEC-bearing mice receiving ZN8. (e) Statistically significant from SEC-bearing mice receiving 5-FU.

The expression level of miRNA-191-5p was upregulated in the SEC control group by 3-fold in comparison with the normal group as shown in Figure 7. On the other hand, the miRNA-191-5p expression level in MET, ZN8, and 5-FU treated animals showed significant downregulation by 20.4%, 57.5%, and 34.8%, respectively, compared to the SEC control group with ZN8 treatment producing the most pronounced effect. In contrast, miRNA-543 expression level was markedly downregulated in the SEC control group by 78.7% when compared to the normal control group as shown in Figure 7. Administration of MET, ZN8, and 5-FU produced a significant upregulation in miRNA-543 expression by 2.14-, 3.2-, and 3.87-fold respectively, relative to the SEC control group.

## 3. Discussion

Several trials were conducted to assess the impact of the independent variables. The response surface approach is based on the experimental design that seeks to identify the optimal variables for a certain objective of the response with the fewest number of tests. It is worth mentioning that the EE% of hydrophilic drugs may be relatively small due to their hydrophilic nature [15]. For zein amount (mg) (X_1_), the decrease in EE% values might be related to the nature of both zein and MET (hydrophilic drug), as it was previously reported, interactions between hydrophilic and hydrophobic molecules make it more challenging to encapsulate hydrophilic drugs in ZNs than hydrophobic drugs [8]. In addition to this, hydrophilic drugs favored the formation of macroaggregates and sediments, suggesting destabilization of the colloidal structure [16]. Regarding bile salt amount (mg) (X_2_), increased bile salt amount promotes the MET solubility in the aqueous media by generating mixed micelles and boosting drug EE% into the ZNs. Our findings were consistent with Salem et al. as they prepared MET-loaded bilosomes. EE% values were found to be augmented by increasing the bile salt amount [17].

For bile salt type (X_3_), the increase in EE% values when using SDC as opposed to SC may be explained by the fact that SDC has a surface-active property and capacity to integrate into the bilayer membrane surface, both of which contribute to an increase in the elasticity of the bilayer membrane and the drug solubility into the membrane, thus augmenting the EE%. Moreover, the higher hydrophobicity of SDC (HLB = 16) compared to SC (HLB = 18) served as a barrier to slow the drug leakage from nanoparticles, which led to higher EE% [18]. Salem et al. also observed the fact that employing SDC increased the EE% compared to SC [17].

It is worth mentioning that small nanoparticles enter tumors more evenly than large nanoparticles. These properties may make small particles more suitable drug delivery vectors [19], confirming the excellent capability of ZNs to penetrate tumors successfully. For zein amount (mg) (X_1_), the PS values decreased by increasing the zein amount [20] due to the formation of larger self-assembled ZNs [21]. Regarding bile salt type (X_3_), the higher PS upon using SDC compared to SC was consistent with Joshi et al. through their fabrication of chlorpheniramine maleate proniosomes for transdermal delivery [21]. It is worth noting that ZNs prepared by SDC showed both higher EE% and PS compared to SC, as it was previously reported by Hathout et al. that there is a direct relationship between the increase in EE% and PS augmentation [22]. ZP is widely used to evaluate the charge of nanoparticles, serving as an excellent predictor of the stability of nanoparticles [23]. Increasing the zein amount (mg) (X_1_) significantly decreased ZP due to reports that zein is a positively charged polymer [24], hence we speculate that by increasing amounts of zein, absolute values of ZP will decrease. ZP values were augmented using SC compared to SDC. The previous findings were in accordance with Abd El-Alim et al. [25] that might be related to the difference in the chemical structure of both SC and SDC as the former contained an extra OH group compared to the latter [26].

The average bias percent values of all acquired responses for the optimum ZNs (ZN8) were less than 10%, indicating the high model’s prediction capabilities [13]. Furthermore, the drug’s release profile is a key predictor of its in vivo performance. Encapsulation of MET within ZNs has resulted in its retention, reducing its release in comparison to its solution. This was demonstrated by its reasonable sustained release profile from ZN8 compared to the MET solution. Two factors might support the preceding result. First, zein contains a significant amount of nonpolar amino acids, which have the potential to combine and entrap drugs by creating stable protein-drug complexes. As a result, it may cause drugs to release more slowly [27]. The second reason is the existence of PC, creating a drug-phospholipid complex loaded inside the ZNs, potentially providing the opportunity to keep combating the tumor cell [28]. According to DSC investigations, there is a significant interaction between the components of ZNs and MET which might account for the high EE% of MET. These interactions may be responsible for good structure, shape, and excellent stability [29].

In the current study, mice implanted with EAC cells developed solid tumors with a gradual tumor volume increase within the following two weeks. Ehrlich carcinoma is a transplantable model for BC that is sensitive to chemotherapy because it is an undifferentiated carcinoma with a high growth rate [30]. Prior research suggests that MET may lower cancer incidence and cancer-related mortality in diabetic patients [31].

The present results revealed that treatment of SEC mice with the optimum MET-loaded ZNs (ZN8) improved the survival rate and decreased the tumor volume of SEC mice. These outcomes were in line with a number of in vivo studies that showed that MET can prevent the growth of the colon, pancreatic, and prostate carcinoma cells effectively [32,33,34].

The antitumor effect of MET and ZN8, as detected in this study, was accompanied by an elevated P53 level and the induction of apoptosis, which was more pronounced in ZN8-treated mice. Chemotherapeutic drugs’ primary targets include upregulating and activating P53. Upon activation, P53 inhibits tumor progression through restriction of the growth and survival of tumor cells. The growth and progression of tumor mass may be facilitated by P53 or P53 pathway mutations [35]. Several studies have found that P53 is involved in the anticancer activity of MET [36,37]. It was found that MET activated AMPK, which in turn promoted P53 phosphorylation and activation, inhibiting melanoma invasion and metastasis [37]. Other research found that MET and 2-deoxyglucose together stimulate apoptosis in prostate cancer cells in a P53-dependent manner [36]. Furthermore, it was reported that MET-induced growth inhibition, senescence, and apoptosis in BC cells depends on P53 [38,39].

In the current study, the level of the active phosphorylated form of AMPK was significantly increased by ZN8 treatment. This is in agreement with several previous studies reporting that MET is hypothesized to have an anti-proliferative impact via an AMPK-dependent mechanism [40,41]. The proliferation of epithelial cells and overall mRNA translation and protein synthesis may be reduced when AMPK is activated, as in BC tissue [42]. A former study on MET’s impact on BC demonstrated that it inhibited cell proliferation via targeting the AMPK signaling pathway [43].

It is widely known that AMPK promotes factors such as peroxisome proliferator–activated receptor γ coactivator 1α (PGC-1α), P53, Sirtuin 1 (SIRT1), and Forkhead box O (FoxO), all of which can block the NF-κB signaling via distinctive mechanism [44]. Since ZN8 in the current study augmented the active form of AMPK the most, we may hypothesize that the lower level of NF-κB is a result of the activation of AMPK-related pathways.

The chemotherapeutic effect of MET (and in particular ZN8) was related to a significant decrease in cyclin D1 level in the present work. This is consistent with the findings of Lengyel et al. [45], who demonstrated that MET inhibited ovarian cancer progression in animal models by halting the cell cycle at G0/G1 that was associated with a reduction in cyclin D1 levels. Another study reported that MET was able to reduce cell viability and inhibited the rate of colony formation in BC cells. The higher proportion of cells in the G0/G1 phase and lower levels of cyclin D1 were indicators of anticancer effects of MET which were also accompanied by cell cycle arrest [46].

In this study, MET and ZN8 produced a significant elevation in the apoptotic marker caspase-3 level. This is in agreement with a previous study reporting the induction of caspase-dependent cell death by MET in BC cells [47]. In a different study, high doses of MET administration enhanced the antitumor activity of carboplatin evidenced by an increase in the pro-apoptotic activators caspase-3 and bax [48].

The outcomes of the current work showed the pronounced effect of ZN8 and MET treatment of SEC mice in the reduction of both COX-2 and PGE2 levels. This is in accordance with a recent study revealing that MET inhibited COX 2 and PGE2 expression in BC cells in a time and dose dependent manner. Moreover, MET was found to regulate COX 2 production at each of the transcriptional and post transcriptional levels [46]. It has been reported that MET decreases PGE2 synthesis via AMPK activation [49], and COX 2 is the primary enzyme in PGE2 synthesis which establishes a potential association between AMPK activation by MET and suppression of inflammatory events. Therefore, it is hypothesized that MET may play an anticancer role via the AMPK mediated COX 2 signaling pathway which may alter cell cycle progression to control the development of tumors.

The potential role of MET and in particular ZN8 in the modulation of tumor-associated miRNAs as part of the novel mechanism of action of MET as an anticancer agent in the development and progression of BC was investigated in the current study. MET has been reported to alter miRNAs levels in the management of a variety of diseases via AMPK-dependent or AMPK-independent mechanisms [50].

In the present work, miRNA-191-5p expression level was upregulated in SEC mice. This is in agreement with a previous study reporting overexpression of miR-191-5p in BC cells contributes to a small number of apoptotic bodies and a reduction in caspase-3/-7 activity as well as reduced P53 expression [51]. In addition, miRNA-191 was found to promote tumorigenesis of human colorectal cancer [52]. Both MET and ZN8 treatment of SEC mice produced a marked downregulation of miRNA-191-5p expression level. On the contrary, miR-543 expression level was markedly upregulated in both MET and ZN8 treated mice. Chen et al. previously demonstrated that miR-543 inhibited BC cell proliferation, hindered cell cycling, and accelerated cell apoptosis [53].

## 4. Materials and Methods

### 4.1. Materials

Sodium cholate (SC), sodium deoxycholate (SDC), L-a phosphatidylcholine (PC), and zein were purchased from Sigma Aldrich (Burlington, MA, USA). Chloroform, ethanol, and methanol were purchased from El-Nasr Pharmaceutical Company, (Cairo, Egypt). Metformin hydrochloride (MET) was received as a kind gift from the SEDICO pharmaceutical industries (Cairo, Egypt).

### 4.2. Fabrication of MET-Loaded ZNs

ZNs were fabricated applying ethanol injection method with some modifications. Firstly, PC (100 mg) with (SDC or SC) and zein at different concentrations were weighed and then dissolved in 2 mL of ethanol and chloroform mixture (1:1 *v*/*v*) (Table 5). MET (20 mg) was dissolved in 10 mL distilled water and then injected into the lipophilic mixture and stirred using a magnetic stirrer (Model MSH-20D, GmbH, Berlin, Germany) at a temperature of 25 °C at 1500 rpm for 30 min for complete solvent evaporation. To enhance the particles’ homogeneity in dispersion media, a probe sonicator (JY-92-II, Xinzhi, China) was utilized to sonicate the fabricated dispersion for 5 min (3 s on and 3 s off) at 40% amplitude. Finally, the formulae were stored in a refrigerator for maturation [54].

### 4.3. In Vitro Characterization and Optimization of MET-Loaded ZNs

#### 4.3.1. Calculation of Entrapment Efficiency Percent (EE%)

ZNs were centrifuged at 20,000 rpm for 1 h at 4 °C applying a cooling centrifuge (Sigma 3K 30, Steinheim, Germany) [55]. Centrifugation was followed by methanol lysis of the sediment, which was then subjected to analysis at 233 nm [56] applying a UV-Vis spectrophotometer (Shimadzu UV 1650 Spectrophotometer, Tokyo, Japan). The equation used to calculate MET EE% is as follows [57]:

EE% = ((entrapped MET))/(total MET concentration)(1)

#### 4.3.2. Calculation of Particle Size, Polydispersity Index, and Zeta Potential

Zetasizer (Nano ZS, Malvern Panalytical Ltd., Malvern, UK) was utilized to assess the PS and PDI. Prior to the measurement, the formulae were diluted. Utilizing the same equipment, ZP was assessed via tracking the mobile particles in an electric field [58,59,60].

#### 4.3.3. Optimization of ZNs

D-optimal design applying Design Expert^®^ software (Version 7, State-Ease Inc., USA) was used to obtain the optimum formula using the desirability criterion and in compliance with the constraints provided in Table 5. Zein amount (X_1_), bile salt amount (X_2_), and bile salt type (X_3_) were explored as independent variables, while EE% (Y_1_), PS (Y_2_), and ZP (Y_3_), were selected as dependent variables. This formula was fabricated and characterized. The recorded, predicted, and observed responses were then linked applying the following equation to get the bias percent [61].

Bias percent = ((predicted value − observed value))/(observed value) × 100(2)

#### 4.3.4. Transmission Electron Microscope (TEM)

Morphology of the optimum ZNs formulation was observed via TEM (JoelJEM 1230, Tokyo, Japan). On a copper grid with carbon laminate, one drop of the optimum formulation was placed as a thin layer and dyed with 1.5% phosphotungstic acid [13].

#### 4.3.5. In Vitro Release Study

USP dissolution apparatus II (Pharma Test, Hainburg, Germany) was utilized to assess in vitro drug release for 6 h at 37 °C. An amount of 1 mL of the optimum ZNs formulation was put into tubes with 0.706 cm^2^ permeation area with one end firmly sealed with a cellulose membrane and the other end linked to the shaft of the dissolution equipment rather than the baskets. The receptor medium was 50 mL of phosphate buffer saline solution (pH 7.4). Aliquots were withdrawn at 1, 2, 3, 4, 5, and 6 h. The samples were examined applying a UV spectrophotometer set to λmax 233 nm. The measurements were carried out in triplicate ± SD [62].

#### 4.3.6. Effect of Short-Term Storage

For a period of three months, the optimum ZNs formulation was kept at 4–8 °C to test its physical stability. It was tested for visual, EE%, PS, PDI, and ZP alterations before and after storage. The gathered data were statistically examined utilizing the Student’s *t*-test in SPSS^®^ program 22.0 [63,64].

#### 4.3.7. Differential Scanning Calorimetry

Differential scanning calorimetry (DSC-60, Shimadzu Corp., Kyoto, Japan) that was indium-standardized was utilized to determine the thermal properties of MET and the optimum ZNs. In an aluminum pan, 5 mg of samples were heated at a rate of 5 °C/min under a nitrogen stream (25 mL/min) at a temperature range of 10–250 °C [65].

### 4.4. In Vivo Study

#### 4.4.1. Animals and Experimental Design

The current investigation was carried out in accordance with the guidelines for the care and use of laboratory animals approved by the Research Ethical Committee, Faculty of Pharmacy, October 6 University, Egypt (PRE-Ph-2212046). A total of 70 adult female Swiss Albino mice weighing 20–25 g were acquired from the National Research Center (Cairo, Egypt). For acclimatization, mice were kept for a week and fed a standard pellet diet while being given unlimited access to water. Ehrlich ascites carcinoma (EAC) cells in a predetermined number (1 × 10^6^ cells) were acquired from the National Cancer Institute’s Pharmacology and Experimental Oncology Unit at Cairo University in Giza, Egypt. EAC cells were injected into the peritoneal cavity of a mouse and allowed to proliferate. In 10 days, an ascitic fluid containing Ehrlich tumor cells was formed, removed with a sterile syringe, diluted in 0.9% sterile saline (1:9 *v*/*v*), and measured with a Neubauer Hemocytometer (Sigma Aldrich, St. Louis, MO, USA). Viable EAC cells (2.5 × 10^6^ cells) were injected subcutaneously into the right thigh of the lower limb of mice to induce solid Ehrlich carcinoma (SEC). A solid tumor mass (about 300 mm^3^) became palpable after 12 days.

Animals were divided into the following five groups: Group (I): Control normal non-tumor bearing mice, received vehicle (*n* = 10); Group (II): SEC control (control tumor bearing mice, received vehicle (*n* = 15)); Group (III): SEC bearing mice + MET (20 mg/kg) [66] (*n* = 15); Group (IV): SEC bearing mice + optimum ZNs formulation (20 mg/kg) (*n* = 15). Both groups III and IV were injected i.p. for four cycles every five days (on the 12th, 17th, 22nd, and 27th days). Group (V): SEC bearing mice + 5-FU (20 mg/kg) [67] injected i.p. on alternate days from the 12th day till the end of the experiment (*n* = 15).

The survival rate for each experimental group was calculated applying this formula: survival rate = (number of live animals in a group on the 28th day/total number of animals in the same group at the start of the experiment) × 100 [68]. The tumor’s dimensions were assessed using a vernier caliper (Tricle Brand, Shanghai, China) beginning on the 12th day and subsequently, every 2 or 3 days till the experiment ended. The following formula was employed to determine the tumors’ volume: tumor volume (mm^3^) = 0.52 AB^2^, where A is the length of the minor axis and B is the length of the major axis [69]. According to Salem et al., the tumor inhibition rate (TIR) was assessed applying this formula: TIR = ((mean tumor volume of control tumor group − mean tumor volume of the treated group) × 100/mean tumor volume of control tumor group) [70]. Mice were anesthetized with ether on the 28th day before being sacrificed through cervical dislocation.

#### 4.4.2. Assessment of Biochemical Parameters

Tumor tissue was removed and separated into two portions. One portion was stored at −80 °C until RNA was extracted for subsequent RT-qPCR of P53, NF-κB, miRNA-191-5p, and miR-543. The second portion was homogenized, separated into several aliquots, and kept at −80 °C. One aliquot was centrifuged at 1000× *g* and utilized to determine the levels of PGE2 and COX-2 applying enzyme-linked immunosorbent assay (ELISA) kits (Abcam, Cambridge, UK). Another aliquot of tumor homogenate was centrifuged for 15 min at 12,000× *g* and 4 °C to determine the levels of cyclin D1, pAMPKand caspase-3 using ELISA kits provided by Novus Biologicals, LLC (Centennial, CO, USA), Assay Genie (Dublin, Ireland) and Wuhan Fine Biotech Co., (Wuhan, China), respectively.

#### 4.4.3. Real-Time Polymerase Chain Reaction (RT-PCR) for P53, NF-κB, miRNA-191-5p, and miRNA-543

Using the TRIzol technique (Life Technologies Corp., Grand Island, NY, USA), total RNA was isolated from tumor tissue in accordance with the manufacturer’s protocol and stored at −80 °C for further analysis. To evaluate RNA concentration and purity, the Nanodrop 2000 spectrophotometer (NanoDrop Technologies, Wilmington, DE, USA) was used. Using cDNA archive kit (Applied Biosystems, Foster City, CA, USA), RNA was transformed into its complementary DNA. cDNA was utilized for quantitative PCR through SYBR Green (SYBR^®^ Premix Ex Taq™ II, TaKaRa, Dalian, China). Using a 7500 Real-Time PCR System (Applied Biosystems, Foster City, CA, USA), the RT-PCR protocol included an initial denaturation step at 94 °C for 3 min, then 40 cycles for 45 s at 94 °C, then at their respective annealing temperatures for 30 s, and at 72 °C for 30 s followed by a 10-min extension step at 72 °C [71]. The expression of β-Actin served as the internal control, and the relative expression was determined [72]. The primer sequences of tested P53, NF-κB, miRNA-191-5p, and miR-543 as well as β-Actin are displayed in Table 6.

#### 4.4.4. Statistical Analysis

One-way analysis of variance (ANOVA) was used to statistically examine group differences, followed by Tukey–Kramer multiple comparison tests. GraphPad Prism version 7.0 (GraphPad Software, San Diego, CA, USA) was used for all statistical comparisons. All results were presented as mean ± SEM. A difference with *p* < 0.05 was regarded as statistically significant.

## 5. Conclusions

The present research included the preparation of ZNs as novel nanoparticles containing MET as anticancer agent to treat BC. MET-loaded ZN formulations were fabricated using ethanol injection technique. D-optimal design was employed to evaluate the outcomes and conclude the optimum ZN formulation (ZN8) that had high EE%, small PS, and spherical morphology. ZN8 also revealed good stability along with a sustained release profile compared to MET solution. Moreover, the in vivo study revealed that ZN8 treatment of SEC-bearing mice exhibited greater effectiveness when compared to MET. ZN8 formulation produced a marked anti-tumorigenic effect and improved survival rates while correcting the proliferation/apoptosis imbalance. This effect was mediated by the upregulation of tumor suppressor gene P53 and miRNA-543 while decreasing cyclin D1 level and miRNA-191-5p expression. Caspase-3 and pAMPK levels as well as NF-κB gene expression were modulated so that they triggered apoptotic pathway rather than proliferative one. The use of ZN8 enhances MET’s cytotoxic effects through affecting different molecular targets.

## Figures and Tables

**Table 1:** Results of regression analysis for responses (Y_1_) EE%, (Y_2_) PS, and (Y_3_) ZP.

	Model	Adequate Precision	R^2^	Adjusted R^2^	Predicted R^2^	*p* Value
Y_1_: EE%	Quadratic	51.973	0.99	0.99	0.98	<0.0001
Y_2_: PS (nm)	2FI	13.192	0.92	0.86	0.79	0.0005
Y_3_: ZP (mV)	2FI	13.634	0.92	0.86	0.79	0.0005
			EE%	PS (nm)	ZP (mV)	
Predicted values of ZN 8			78.15	55.97	−23.29	
Observed values of ZN 8			77.68	59.69	−24.00	
Bias %			0.60	6.23	2.95	

Abbreviations: EE%: entrapment efficiency percent, PS: particle size, ZP: zeta potential.

**Table 2:** Experimental runs, independent variables, measured responses of MET-ZNs ^a^.

	Zein Amount (mg)	Bile Salt Amount (mg)	Bile Salt Type	EE%	PS (nm)	PDI	ZP (mV)
F1	10	15	SDC	38.77 ± 6.84	126.80 ± 2.46	0.43 ± 0.08	−22.80 ± 3.23
F2	5	15	SDC	43.53 ± 3.90	79.00 ± 8.35	0.76 ± 0.06	−26.90 ± 1.06
F3	10	30	SDC	52.55 ± 1.07	103.00 ± 2.98	0.73 ± 0.01	−21.40 ± 0.61
F4	10	30	SDC	52.55 ± 1.07	103.00 ± 2.98	0.73 ± 0.01	−21.40 ± 0.61
F5	5	30	SDC	77.68 ± 1.28	59.69 ± 1.79	0.49 ± 0.03	−24.00 ± 0.80
F6	7.5	22.5	SDC	32.75 ± 7.19	71.51 ± 22.90	0.34 ± 0.12	−23.86 ± 1.23
F7	7.5	15	SDC	40.31 ± 2.60	121.90 ± 3.86	0.99 ± 0.01	−23.20 ± 1.26
F8	5	30	SDC	77.68 ± 1.28	59.69 ± 1.79	0.49 ± 0.03	−24.00 ± 0.80
F9	5	15	SC	57.73 ± 1.39	87.33 ± 3.60	0.43 ± 0.01	−26.70 ± 0.55
F10	8.75	22.5	SC	37.39 ± 12.10	73.91 ± 1.17	0.51 ± 0.08	−23.90 ± 0.51
F11	5	22.5	SC	28.79 ± 6.54	69.21 ± 2.83	0.54 ± 0.11	−24.10 ± 2.42
F12	10	15	SC	40.78 ± 4.91	64.96 ± 1.95	0.36 ± 0.05	−20.30 ± 0.32
F13	10	30	SC	40.91 ± 7.17	62.55 ± 1.14	0.41 ± 0.01	−21.40 ± 1.73
F14	10	30	SC	40.91 ± 7.17	62.55 ± 1.14	0.41 ± 0.01	−21.40 ± 1.73
F15	7.5	15	SC	32.26 ± 9.97	62.33 ± 1.33	0.40 ± 0.01	−20.10 ± 2.46

Presented values are the mean ± SD (*n* = 3). ^a^ All formulae contained 20 mg MET and 100 mg phosphatidylcholine in a volume of 10 mL. Abbreviation: MET: metformin; SAA: surfactant; SC: sodium cholate; SDC: sodium deoxycholate; EE%: entrapment efficiency percent; PS: particle size; PDI: polydispersity index; ZP; zeta potential, and ZNs; zein nanoparticles.

**Table 3:** Effect of MET and ZN8 treatment of SEC bearing mice on tumor P53, NF-κB gene expression, and pAMPK level.

No.	Group	P53 (Relative Expression)	NF-κB (Relative Expression)	pAMPK (ng/g Tissue)
I	Normal control	1.01 ± 0.026	1.08 ± 0.022	10.73 ± 0.28
II	SEC control	0.67 ± 0.034 ^a,c,d,e^	3.53 ± 0.11 ^a,c,d,e^	5.61 ± 0.32 ^a,c,d,e^
III	SEC bearing mice + MET	1.15 ± 0.055 ^b,d,e^	2.95 ± 0.046 ^a,b,d,e^	8.07 ± 0.39 ^a,b^
IV	SEC bearing mice + ZN8	4.78 ± 0.088 ^a,b,c,e^	1.61 ± 0.027 ^a,b,c,e^	9.06 ± 0.78 ^b^
V	SEC bearing mice + 5-FU	3.22 ± 0.059 ^a,b,c,d^	2.41 ± 0.033 ^a,b,c,d^	9.57 ± 0.48 ^b^

Notes: pAMPK protein level is expressed as mean ± SEM. Gene expression levels of P53 and NF-κB are expressed as relative quantification mean ± SEM using β-actin as reference gene. The data were analyzed using one way ANOVA followed by Tukey’s multiple comparisons test. A difference with *p* < 0.05 was considered to be statistically significant. ^a^ statistically significant from normal controls. ^b^ statistically significant from SEC control. ^c^ statistically significant from SEC bearing mice receiving MET. ^d^ statistically significant from SEC bearing mice receiving ZN8. ^e^ statistically significant from SEC bearing mice receiving 5-FU. NF-κB: Nuclear factor kappa B, pAMPK: Phosphorylated AMP-Activated Protein Kinase, SEC: Solid Ehrlich carcinoma, MET: metformin, ZN8: optimum metformin loaded zein nanoparticles, 5-FU: 5-flourouracil.

**Table 4:** Effect of MET and ZN8 treatment of SEC bearing mice on tumor cyclin D1, caspase-3, COX-2 and PGE2 levels.

No.	Group	Cyclin D1 (ng/g Tissue)	Caspase-3 (pg/g Tissue)	COX-2 (U/mg Tissue)	PGE2 (pg/g Tissue)
I	Normal control	0.585 ± 0.026	11.27 ± 0.293	23.2 ± 0.928	103.7 ± 3.83
II	SEC control	4.37 ± 0.165 ^a,c,d,e^	0.87 ± 0.073 ^a,c,d,e^	82.10 ± 2.66 ^a,c,d,e^	924.5 ± 15.89 ^a,c,d,e^
III	SEC bearing mice + MET	3.11 ± 0.157 ^a,b,d,e^	3.15 ± 0.124 ^a,b,d,e^	63.56 ± 2.81 ^a,b,d,e^	631.5 ± 17.02 ^a,b,d,e^
IV	SEC bearing mice + ZN8	1.42 ± 0.075 ^a,b,c,e^	5.4 ± 0.148 ^a,b,c,e^	42.84 ± 1.52 ^a,b,c,e^	232.9 ± 7.21 ^a,b,c,e^
V	SEC bearing mice + 5-FU	0.853 ± 0.063 ^b,c,d^	7.12 ± 0.274 ^a,b,c,d^	32.73 ± 1.11 ^a,b,c,d^	151.4 ± 4.78 ^a,b,c,d^

All data are expressed as mean ± SEM. The data were analyzed using one way ANOVA followed by Tukey’s multiple comparisons test. A difference with *p* < 0.05 was considered to be statistically significant. ^a^ statistically significant from normal controls. ^b^ statistically significant from SEC control. ^c^ statistically significant from SEC bearing mice receiving MET ^d^ statistically significant from SEC bearing mice receiving ZN8 ^e^ statistically significant from SEC bearing mice receiving 5-FU. COX-2: cyclooxygenase-2, PGE2: Prostaglandin E2, SEC: Solid Ehrlich carcinoma, MET: metformin, ZN8: optimum metformin loaded zein nanoparticles, 5-FU: 5-flourouracil.

**Table 5:** D-optimal design used for optimization of zein nanoparticles.

Factors (Independent Variables)	Factor Type	Levels		
		(−1)		(+1)
X_1_: Zein amount (mg)	Numeric	5		10
X_2_: Bile salt amount (mg)	Numeric	15		30
X_3_: Bile salt type	Categoric	SDC		SC
Responses (dependent variables)		Desirability Constraints		
Y_1_: EE%		Maximize		
Y_2_: PS (nm)		Minimize		
Y_3_: ZP (mV)		Maximize (as absolute value)		

Abbreviation: SDC: sodium deoxycholate; SC: sodium cholate; EE%: entrapment efficiency percent; PS: particle size; ZP: zeta potential.

**Table 6:** Primers used in RT-PCR.

Gene	Primer Sequence	Amplicon Size
p53	F: 5′-AGTCTAGAGCCACCGTCCA-3′ R: 5′-TCTGACGCACACCTATTGCAAGC-3′	443
NF-κB	F: 5′-GCTCAAGATCTGCCGAGTAAA-3′ R: 5′-GTCCCGTGAAATACACCTCAA-3′	113
miR-191-5p	F: 5′CAACGGAATCCCAAAAGCAGCTG-3′ R: 5′TGTCGTGGAGTCGGC-3′	60
miR-543	F: 5- GGAAACATTCGCGGTGC-3′ R: 5-GTGCGTGTCGTGGAGTCG-3′	59
β-Actin	F:5′-GTAGCCATCCAGGCTGTGTTG-3′ R: 5′-TGCCAGTGGTACGACCAGAG-3′	52

## Data Availability

The data presented in this study are available in article.
